# Health Effects and Mechanisms of Inulin Action in Human Metabolism

**DOI:** 10.3390/nu16172935

**Published:** 2024-09-02

**Authors:** Jaime Alonso-Allende, Fermín I. Milagro, Paula Aranaz

**Affiliations:** 1 Department of Nutrition, Food Science and Physiology, Faculty of Pharmacy and Nutrition, University of Navarra, 31008 Pamplona, Spain; jalonsoallende@unav.es; 2 Center for Nutrition Research, University of Navarra, 31008 Pamplona, Spain; paranaz@unav.es; 3 Navarra Institute for Health Research (IdiSNA), 31009 Pamplona, Spain; 4 Centro de Investigación Biomédica en Red de la Fisiopatología de la Obesidad y Nutrición (CIBEROBN), Instituto de Salud Carlos III, 28029 Madrid, Spain

**Keywords:** inulin, SCFA, microbiota, obesity, bifidobacteria, pathway, health, insulin

## Abstract

Inulin is a plant polysaccharide which, due to its chemical structure, is not digestible by human gut enzymes but by some bacteria of the human microbiota, acting as a prebiotic. Consequently, inulin consumption has been associated with changes in the composition of the intestinal microbiota related to an improvement of the metabolic state, counteracting different obesity-related disturbances. However, the specific mechanisms of action, including bacterial changes, are not exactly known. Here, a bibliographic review was carried out to study the main effects of inulin on human metabolic health, with a special focus on the mechanisms of action of this prebiotic. Inulin supplementation contributes to body weight and BMI control, reduces blood glucose levels, improves insulin sensitivity, and reduces inflammation markers, mainly through the selective favoring of short-chain fatty acid (SCFA)-producer species from the genera *Bifidobacterium* and *Anaerostipes*. These SCFAs have been shown to ameliorate glucose metabolism and decrease hepatic lipogenesis, reduce inflammation, modulate immune activity, and improve anthropometric parameters such as body weight or BMI. In conclusion, the studies collected suggest that inulin intake produces positive metabolic effects through the improvement of the intestinal microbiota and through the metabolites produced by its fermentation.

## 1. Introduction

### 1.1. Inulin Structure and Properties

**Figure 1:**
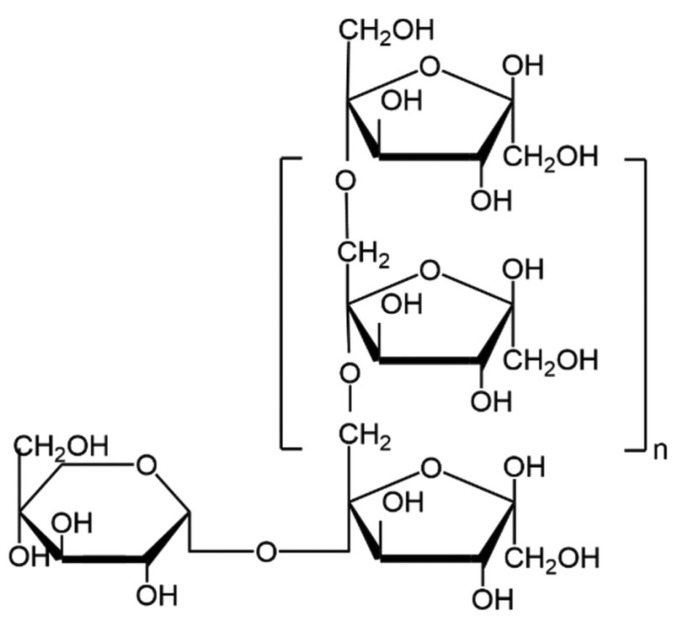
Haworth projection of inulin molecule chemical structure [3].

Inulin is a linear fructan consisting of fructosyl units linked by β (2→1) bonds, typically with a glucose moiety attached at the end through an α (1→2) linkage [1]. Structurally, it is a polysaccharide made up of D-fructofuranose units, rendering it resistant to hydrolysis by human gastrointestinal enzymes due to its β-configuration (Figure 1) [1]. The molecular formula GFn denotes the presence of a terminal glucose unit (G) and fructose units (F), with ‘n’ indicating the number of fructose units [2].

Depending on the degree of polymerization (DP), ranging from 2 to 60, inulin can be categorized into two main varieties: short-chain inulin, containing 2–10 fructose units, and long-chain inulin, containing 10–60 fructose units [2,3]. The DP determines inulin’s properties (viscosity, solubility or even color) and it is influenced by several factors such as plant maturity, climate, and extraction techniques [1,3]. The DP also affects the organoleptic properties of inulin, the short-chain form being sweeter than the long-chain form, which is why it is often that these varieties are commonly used as a sucrose substitute and as a fat substitute, respectively [3]. In addition, its chemical structure, which contains fructose monomers with anomeric C2 in beta-configuration, makes it resistant to hydrolysis by digestive enzymes, making it a non-digestible carbohydrate for humans [2,4]. Consequently, inulin has been principally studied and used as a prebiotic, as it can be digested by some bacteria of the human microbiota and can have positive health effects, particularly in obesity-related metabolic diseases [2,3].

### 1.2. Sources of Inulin

Inulin is a polysaccharide that can be found in a wide variety of plant families, with *Liliaceae*, *Amaryllidaceae*, and *Asteraceae* being its main natural sources [2,5]. Inulin is stored in different regions of the plant, including bulbs, roots, and tubers, depending on the needs and physiology of each species [5]. In terms of species, chicory (*Cichorium intybus* L.) is a major natural source of inulin, containing approximately 20% inulin by wet weight and 80% by dry weight in the roots [6]. Chicory inulin presents the highest fructose/glucose ratio [7]. Jerusalem artichoke (*Helianthus tuberosus*), which belongs to the *Asteraceae* family, also contains a high amount of inulin (17–20% by wet weight) and stores it in its tubers [8]. Garlic (*Allium sativum* L.) stores inulin in its bulb and has similar content as chicory, around 75% by dry weight [3].

In addition, since inulin’s main function is to store energy as carbohydrates, the time in the plant’s life cycle at which inulin is extracted must be considered. This determines the degree of polymerization, since it is increased or reduced according to energetic and physiological needs. Roughly speaking, the degree of polymerization increases from spring to mid-autumn and declines with the onset of winter until the following spring. Therefore, even if two inulin samples are taken from the same plant, they can be very different if they are collected at two different stages of the life cycle.

### 1.3. Study Aim

The present article aims to review the effects of inulin on obesity and human microbiota by compiling the results obtained in the clinical trials available to date that have investigated the effect of inulin on both aspects of human health. Furthermore, this study tries to emphasize the mechanisms of action that justify its use in obesity and metabolic syndrome.

## 2. Materials and Methods

**Figure 2:**
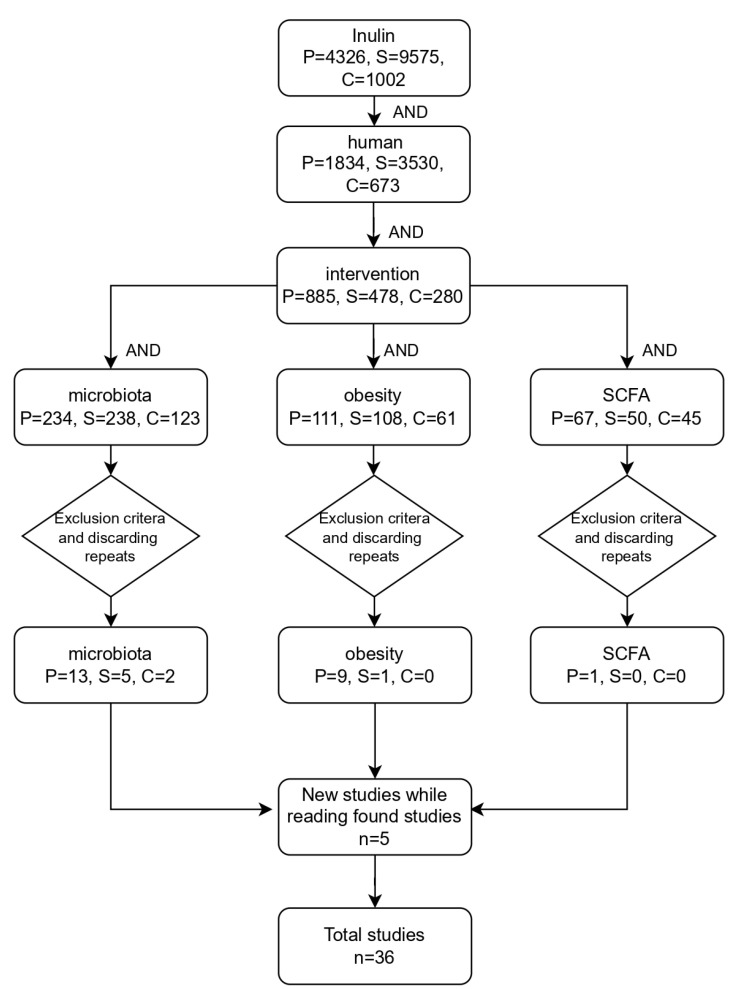
Flowchart depicting the search strategy. P: PubMed; S: Scopus; C: Cochrane Library.

This article is based on the available literature in MEDLINE (PubMed), Scopus, and Cochrane Library to identify clinical trials related to the effect of the prebiotic inulin on human microbiota and obesity. The study encompasses all full-text publications that were available between January 2009 and 28 June 2024. The search strategy is shown in Figure 2.

Exclusion criteria were settled for the purpose of filtering the studies obtained, which included studies without human subjects, studies with the main topic of the study not related to the research, studies using symbiotics without specifying the inulin content, studies with a low number of human subjects (below 10) or short study duration, and those studies where only the abstract was available. For the analysis of the results, the following parameters were considered in each article: type of study, population, cohort, patient status, age, sample size, sex (M/F), inulin origin, dose, characterization, characterization control, duration (weeks), intervention group, control group, increasing effect, decreasing effect, no effect, inulin mechanism of action, and adverse effects.

## 3. Study Characteristics

The studies analyzed are listed in Table 1, which describes the general characteristics and the status of their participants.

The most common studies analyzed include the implementation of randomized, controlled trials (RCTs) and various combinations of blinding and control measures such as double-blinding, placebo control, and crossover designs. Conversely, characteristics such as single blinding, multicentric settings, and specific population focuses like obese individuals appear less frequently.

Among the countries where these studies have been conducted, the most common ones include Belgium, the United States, and the United Kingdom. Additionally, Canada, Spain, Germany, and Italy are mentioned multiple times, indicating their significant contribution to the body of research in this field. Comparatively, it must be emphasized that there are very few studies from Asia and none from Africa or South America. These findings are conclusive, as the geographical distribution of the studies coincides with the biodistribution of the main natural sources of inulin, such as chicory or Jerusalem artichoke. However, it is noteworthy that countries with higher numbers of studies also tend to have better socioeconomic conditions.

Moreover, there is a notable diversity in the origins of inulin utilized in the analyzed studies. The most common source of inulin in the analyzed studies is chicory, including other natural sources such as Jerusalem artichoke and agave, whereas some studies do not specify their inulin origin. Additionally, specific commercial brands are also observed, where Beneo Orafti stands out as the main supplier.

The intervention groups consisted of a variety of treatments involving inulin or an inulin-containing compound, such as inulin–propionate ester or combinations of inulin with other substances like maltodextrin or polyphenols [9,18,23]. In addition, the administration form varies widely, including powder, snack, chews, and beverages [18,20,21,27,41,43]. Notably, inulin alone is frequently used as an intervention across multiple studies, appearing multiple times as a treatment group. On the other hand, the control groups typically involve the administration of maltodextrin as placebo, as it is very similar to inulin in taste and flavor. However, glucose, cellulose, and even fruits are sometimes used as well [9,12,27,29,41,43]. Control groups also include baseline measurements or control diets without the intervention substance. Among the provided doses of inulin, the range varies from 3 g per day to 20 g per day.

Most of the analyzed studies include adults with overweight or obesity [9,12,18,19,23,24,27,42]. Additionally, healthy adults represent a prevalent group [15,16,20,21,28,31,33,34,37,39,41,43]. Specific medical conditions such as type 2 diabetes mellitus and obesity-related metabolic disorders also happen to appear, indicating a focus on investigating interventions in these populations [14,17,25]. Notably, there is a significant representation of studies involving children, particularly those with obesity-related conditions like obese children with elevated body mass index (BMI) or healthy children with overweight and obesity [10,11,44]. Furthermore, certain studies target specific groups such as patients with hypertrygliceridemic and hypercholesterolemic status, chronic kidney disease, or celiac disease [22,30]. Overall, these findings reflect a diverse range of patient populations targeted in clinical research, spanning from healthy adults to individuals with various medical conditions across different age groups.

Among the age ranges utilized in the analyzed studies, a broad spectrum is observed, with the most common range being adults aged 18 to 65 years old [19,23,24,27,28,37,38,41,42,43]. This range encompasses a significant portion of the research population, indicating a focus on adult participants across various studies. Additionally, certain studies targeted narrower age groups, such as children aged 7 to 15 or adults aged 40 to 80 [10,11,12,22,29]. For the total number of individuals studied per trial, the highest numbers of individuals studied in a single trial was 174, while the lowest was 12. Above this mean value, (approximately 58) there are 11 trials with participant numbers ranging from 59 to 174, indicating larger-scale studies. Conversely, below the mean value, there are 16 trials with participant numbers ranging from 12 to 49, suggesting smaller-scale studies or trials with more specific inclusion criteria. Regarding the male/female ratio, the highest male/female ratio is 33/11, while the lowest ratio is 0/30 [13,19]. Overall, while the differences may not be statistically significant, there is a slight tendency towards a higher proportion of men studied, being predominant on 16 of the analyzed trials.

## 4. Inulin Effect on Human Metabolic Health

The studies compiled on the role of inulin on metabolic health are listed in Table 2, which specifies the physiological effects and its activity modulating gut microbiota.

### 4.1. Physiological Effects of Inulin

All inulin physiological effects are detailed in Table 2. Several human studies have shown that inulin supplementation leads to a significant improvement in anthropometric parameters, particularly body weight and BMI. In fact, two independent clinical trials found that 16 g/d of inulin for 12 weeks significantly decreased both parameters in adults with obesity or metabolic disorder, even though 10 g/d for 8 weeks has been observed to achieve similar results [17,24,35]. However, two studies with similar dosages (21 g/d and 20 g/d) and identical intervention periods (6 weeks) showed contradictory results as they found beneficial or no effects on anthropometric parameters, respectively [9,42]. Connected to this, anthropometric parameters are closely linked to an individual’s metabolic status, such as blood glucose levels or insulin sensitivity, which are key determinants of overall metabolic health. In relation to this, it has been found that inulin supplementation could reduce insulin levels in blood and improve insulin sensitivity [9,24]. For example, inulin consumption at 10 g/d for 8 weeks ultimately ends up lowering blood glucose levels, improving glucose overall metabolism [35]. In addition, it is noteworthy that inulin can achieve these effects within a wide range of dosages and intervention time periods [9,30,35,38]. Regarding other physiological parameters, inulin can positively affect the metabolic lipid profile. Thus, two studies have shown that inulin can reduce LDL-c, total cholesterol, and triglyceride blood levels, even at different doses [30]. However, the use of natural sources seems to be an important factor, since Jerusalem artichoke inulin and chicory inulin showed contradictory effects at similar doses. In addition, inulin is capable of increasing HDL-c serum levels when it is consumed for a long time, whereas short-time consumption has been seen not to be effective [24,30]. Interestingly, inulin supplementation improves inflammation status as it has been observed to decrease proinflammatory cytokines and biomarkers, such as TNF-α or calprotectin, as well as increase anti-inflammatory parameters such as IL-10 [9,25,26].

### 4.2. Link between Microbiota Modulation and Physiological Effects Produced by Inulin Supplementation

As discussed in the introduction, inulin can only be digested by some bacterial species present in the human microbiota, generating changes in its composition that eventually produce the physiological effects observed after inulin supplementation. Among them, most of the studies show that inulin significantly increases different *Bifidobacterium* species, regardless of dose or inulin source, highlighting *B. longum*, *B. adolescentis*, and *B. angulatum* [17,24,29,34]. Surprisingly, inulin was observed not to affect *Bifidobacterium* populations at 20 g/d dose for 6 weeks when following a low-fiber diet or when presenting a metabolic disorder status, suggesting that metabolic status may influence inulin prebiotic action, since 20 g/d dose for 4 weeks in healthy subjects has been found twice to significantly increase *Bifidobacterium* species [15,33,34]. In addition to this, inulin has been found to negatively impact *B. bifidum* at low doses but has an increasing effect at doses over 16 g/d [24]. Nevertheless, it may be possible that this outcome is not caused directly by inulin supplementation but due to competitive exclusion, as other Bifidobacteria species may be better at utilizing inulin as an energy source, which would explain why higher doses positively affect this species [15,32].

This marked bifidogenic effect has been related to different effects on either anthropometric or physiological parameters. Notably, *Bifidobacterium* increase has been associated with higher insulin sensitivity and improved insulin blood levels [17,38]. In addition, the revisions performed suggest that the dosage plays a crucial role in this bifidogenic effect of inulin, modifying its influence over anthropometric parameters. Thus, studies have shown that at high doses administered over a prolonged period, the increase in Bifidobacteria is associated with improvements in these values, especially body weight and BMI [17,24]. Conversely, at lower doses or shorter administration periods, an increase in these species is observed without a corresponding improvement in body measurements [20]. In relation to the lipid profile, the increase in Bifidobacteria following inulin supplementation presents highly contradictory data regarding total cholesterol levels. Two studies using the same source of inulin, at the same dosage, over the same duration and on the same geographical population yielded conflicting results: one study reported an increase in total cholesterol, while the other showed no significant changes [17,24]. These discrepancies highlight the need for further research to elucidate the effects of inulin on lipid profiles.

Although *Bifidobacterium* increase is the most common inulin effect on the intestine, it has also been reported that inulin positively affects the *Anaerostipes* genus independently of dosages or intervention periods. Increases in species of this genus have been related to beneficial physiological effects, including the improvement of glycemia and anthropometric parameters [9]. Specifically, *Anaerostipes* is related to a rapid improvement of inflammatory markers as 6 weeks inulin intervention showed increased anti-inflammatory and decreased proinflammatory markers [9]. On the other hand, inulin’s modulation of the gut microbiota has other positive effects on health since by increasing their number so effectively and lowering the pH of the colon, they hinder and prevent the development of pathogenic or non-beneficial species. For example, inulin-induced microbiota modulation significantly reduces the population number of species belonging to the *Clostridium* genus, leading to reduced inflammation and enhanced overall digestive health and nutrient absorption.

Lastly, it should be clarified that, even though SCFAs increase theoretically, several studies have found no effects on SCFA levels. Nevertheless, as most of the studies analyzed them in fecal samples, it may be possible that enterocytes have consumed most of them, since SCFAs are the main source of energy for them, and therefore, samples containing a lower amount of SCFAs than actually produced were being analyzed.

## 5. Effect of Inulin Combinations with Other Compounds on Human Metabolic Health

The studies compiled on the role of inulin combinations on metabolic health are listed in Table 3, which specifies the physiological effects and its activity modulating gut microbiota.

### 5.1. Effects of Inulin Combinations with Other Compounds on Human Metabolic Health

Different studies have evaluated the effect of supplementation of inulin combined with different compounds or prebiotics in order to find a synergistic effect that enhances the effects produced by inulin alone, which are summarized in Table 3.

Regarding anthropometric measures, inulin improves body weight levels when it is combined with either propionate ester, oligofructose, or green tea catechins [10,12,18]. Among them, regarding dose effect, inulin seems to act more synergistically with oligofructose, as a lower dose of inulin was required to achieve weight improvement when both were combined. Remarkably, catechins accelerate inulin’s beneficial effects, as observed after just 3 weeks of intervention.

On the other hand, inulin combined with propionate ester has been observed to be time dependent, as beneficial effects on weight have been observed at long time intervention periods. However, inulin has not been found to decrease body weight when it is combined with glucose or maltodextrin, suggesting an antagonistic effect between them. Nevertheless, the study that analyzed the effects produced by the combination of inulin and glucose used a very high dose of the latter, which might be considered to affect the results. In relation to this, it has also been observed that inulin–propionate ester can increase GLP-1 and PYY, promoting satiety and reducing ghrelin levels, which decreases the sensation of hunger [12]. However, inulin is incapable of improving satiety hormone levels when combined with high doses of glucose, whereas it still can decrease ghrelin [27].

Regarding glucose metabolism, inulin has been shown to be capable of improving insulin parameters depending on which compound it is combined with. Interestingly, it seems that inulin and oligofructose act antagonistically as no effects on insulin or glucose levels have been observed. In addition, inulin with catechins has been observed to reduce glucose blood levels, but differences in insulin parameters have not been described [18]. Therefore, it may enhance insulin action or improve glucose metabolism by other mechanisms.

Interestingly, the studies collected show that the effect of inulin supplementation on the lipid profile is not maintained when administered in combination with other compounds. However, one of the reasons for this may be that, even if the studied population had obesity or metabolic disorder, they presented a normal lipid profile. Therefore, there would be no significant differences or improvements when presenting baseline levels within the normal health range. Consequently, further investigations are necessary to clarify these results.

### 5.2. Link between Microbiota Modulation and Physiological Effects

In the same way that inulin alone is capable of generating changes in the bacterial proliferation, its combination with other prebiotics modulates gut microbiota in a different way depending on the compound with which it is combined.

Inulin combined with propionate ester, surprisingly, has been observed not to affect bifidobacteria populations, but other genera such as *Bacteroides*, *Anaerostipes,* or *Blautia* were related to improved insulin metabolic parameters. However, no microbiota modulation has been studied to relate gut hormone regulation with inulin–propionate ester supplementation [9,12]. On the other hand, when inulin was combined with oligofructose or maltodextrin, changes in the bifidobacteria population have been found, in accordance with inulin’s observed effects. Moreover, inulin combined with oligofructose has been also found to increase the population of *Faecalibacterium prausnitzii*, which is one of the most common beneficial colon bacteria [10,36]. However, although changes in the microbiota are maintained at different doses and intervention periods of oligofructose–inulin supplementation, the physiological effects are controversial at the anthropometric level, as both improvements in body weight and the absence of a significant effect have been observed [10,36].

Therefore, although inulin appears to have the capacity to act synergistically with other compounds, due to the limited number of studies utilizing inulin in combination with other treatments and even fewer studies examining the modulation of the microbiota alongside physiological effects in patients, it is challenging to correlate alterations in bacterial species with physiological outcomes. This underscores the necessity for further research in this area.

## 6. Effect of Inulin Intake on Other Diseases

The role of inulin in body weight management, glucose metabolism, and inflammatory markers have evidenced the potential use of this fiber to ameliorate obesity-related diseases, including type 2 diabetes and cardiovascular disease. However, due to its prebiotic and anti-inflammatory capacity, supplementation with this ingredient has been evaluated in diseases where the intestinal microbiota and the resulting inflammation play a role in their pathogenesis, such as kidney and inflammatory bowel diseases.

In this regard, the prebiotic activity of inulin supplementation has been investigated for the amelioration of chronic kidney disease (CKD) due to the gut dysbiosis and dysmetabolism that characterize patients with this pathology, which seems to contribute to the progression to CKD-related complications, including cardiovascular disease [45]. Thus, gut microbiota modulation through the administration of prebiotics such as inulin has been suggested as a potential therapeutic target for CKD due to its effect on the profile of circulating blood metabolites [46]. In this regard, Silvia Lai and colleagues observed that supplementation with inulin (19 g/day) with a low-protein diet (LPD) for 6 months was able to modulate gut microbiota, increasing the abundance of Bifidobacteriaceae, accompanied by a reduction of inflammatory circulating markers in patients with CKD [45]. Interestingly, inulin was able to reduce serum insulin and fasting glucose levels and to improve cholesterol metabolism in the participants of the study, suggesting a cardioprotective role of inulin in CKD [30].

The effect of inulin on intestinal health has also been evaluated in different models of intestinal disease, with variable results. Initially, the use of inulin or inulin-enriched products has been proposed to improve intestinal transit and reduce constipation. Thus, a meta-analysis comprising individuals with chronic constipation revealed a positive effect of inulin consumption on bowel function, improving the stool frequency, stool consistency, transit time, and hardness of stool, while no efficacy was shown for pain and bloating [47]. A similar finding was observed in another study, where supplementation with 12 g per day of inulin for 4 weeks improved bowel function in volunteers with chronic constipation [48]. However, the anti-inflammatory activity of inulin and its role contributing to intestinal function is not always observed in individuals with inflammatory bowel disease, including ulcerative colitis (UC) or Crohn’s disease [49]. Studies using inulin as prebiotic for the treatment of chronic intestinal inflammation have shown benefit in animal models of colitis [50]. In the case of humans, the potential use of oligofructose-enriched inulin has been suggested to ameliorate symptoms in individuals with ulcerative colitis [51,52]. However, despite promising data on inulin in gut health, its usefulness in individuals with inflammatory bowel disease is controversial since some individuals report an increase in flatulence and bloating [49]. Therefore, the use inulin in patients with inflammatory bowel disease requires additional studies to demonstrate its convenience.

## 7. Mechanisms of Action

**Figure 3:**
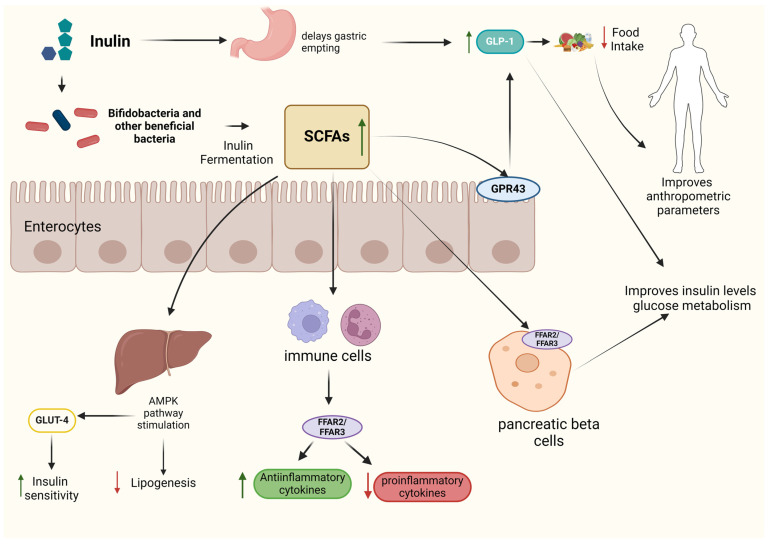
Overview of the primary mechanisms of action and physiological effects of inulin.

The mechanisms of action by which inulin induces beneficial effects in human health are not fully understood. In fact, only a few studies in humans have been designed to explain the underlying mechanisms, which has been a limitation for the present investigation. This is probably due to the fact that, in order to study these mechanisms, the techniques required are excessively invasive. However, we present the proposed mechanisms of action that have been studied in both animal models and in vitro studies that could explain the results observed in humans (Figure 3).

Although inulin primarily exerts its effects through gut microbiota modulation, as previously mentioned, its consumption can also directly influence human physiology. Thus, since it is not digestible, it delays gastric emptying, which increases satiety hormone secretion, such as GLP-1, ending up with a lower food and energy intake and therefore improving anthropometric parameters like body weight [35].

In addition, it also delays glucose absorption in the intestine, helping to regulate the increase in blood glucose levels after meals, favoring glycemic homeostasis [35]. Once inulin arrives in the colon, it is fermented by gut microbiota, principally by *Bifidobacterium* species, even though they represent a low number of the human microbiota, due to several genes which encode for different β-fructofuranosidases, which cleave through inulin structure and produce fermentable oligosaccharides or monosaccharides [53]. Obviously, the ability to utilize inulin varies among species and specific strains of this genus. Those metabolic products are then converted into acetate and lactate by bifidobacteria, which enables cross-feeding interactions between them and SCFA-producing species, such as *Anaerostipes* spp., leading to increased butyrate and propionate levels [53]. In vitro studies have shown that both propionate and butyrate increase GLP-1 and PYY secretion by L-cells via G-protein-coupled receptor 43 (GPR43) human receptor stimulation in a dose-dependent way, regulating food and energy intake [54]. Therefore, depending on the ability of inulin to modulate the gut microbiota, propionate and butyrate would be able to alter GLP-1 and PYY serum levels, which explains why consistent findings are not always found. In addition, hormone secretion regulation has been observed to also impact glucose metabolism, as higher levels of GLP-1 have been observed to improve insulin sensitivity. Therefore, both inulin and inulin-derived SCFAs enhance each other’s effects by similarly altering the secretion of these hormones [54].

SCFAs are able to improve glucose metabolism by other metabolic pathways. In vivo models have found that propionate, butyrate, and especially acetate activate free fatty acid receptor 2 (FFAR2) and FFAR3 receptors in pancreatic beta cells, regulating insulin secretion and improving glucose homeostasis [55,56]. In addition, acetate can improve insulin sensitivity via glucose transporter 4 (GLUT4) by upregulating 5′-AMP-activated protein kinase signaling in liver tissues [57]. Regarding inflammation effects, SCFAs modulate cytokine secretion in a dose-dependent way. It has been reported that SCFAs activate immune cell receptor GPR43 by propionate and acetate or GPR41 by butyrate when they reach a certain concentration level [58,59]. Moreover, it has been suggested that, through the FFAR2 receptor, SCFAs can affect gene expression by inhibiting histone deacetylases and increasing histone acetylation, leading to an increased or decreased secretion of inflammatory cytokines [58]. Related to the lipid profile, acetate and butyrate are used as substrates for lipid formation. However, SCFAs are able to regulate lipogenesis in several ways. In vivo and in vitro studies have shown that activating the AMPK pathway increases the expression of peroxisome proliferator-activated receptor gamma coactivator 1-alpha (PGC-1α), which in turn increases the expression of the transcription factors peroxisome proliferator-activated receptor-α (PPAR-α) and PPAR-γ, which stimulate lipid oxidation [60]. In addition, the AMPK pathway also inhibits transcription factors associated with fat deposition and lipid synthesis.

## 8. Conclusions

According to the collected data, inulin demonstrates significant potential in improving human physiology by acting independently and through modulation of the gut microbiota, particularly species from the *Bifidobacterium* and *Anaerostipes* genera, leading to better anthropometric parameters, improved glucose metabolism, lower insulin levels, and beneficial effects on inflammation and immune function, ultimately improving metabolic states. These positive outcomes have been observed when inulin is used alone or in combination with other compounds, underscoring its versatility and efficacy in promoting metabolic health. However, further research is warranted to fully elucidate its therapeutic potential and optimize its clinical applications.

## Figures and Tables

**Table 1:** Study designs and subjects’ characteristics among the available clinical trials.

Study Type	Inulin Source	Intervention Group	Control Group	Population	Age	Sample Size (Male/Female)	Reference
Randomized, controlled trial	Beneo-Orafti HP, Kreglinger Europe, Antwerpen, Belgium	Inulin-propionate ester	Inulin and Cellulose	English adults with overweight and obesity	18–65	12 (3/9)	[9]
Clinical trial	Oligofructose-inulin (Synergy1; BENEO GmbH, Mannheim, Germany)	Oligofructose-inulin	Maltodextrin placebo	Canadian healthy children with overweight and obesity	7–12	38	[10]
Randomized, double-blind, placebo-controlled trial	Extracted inulin powder from Thai Jerusalem artichoke	Extracted inulin powder	Isocaloric maltodextrin	Thai children with obesity	7–15	165	[11]
Randomized, controlled, cross-over study	Not available	Inulin-propionate ester	Inulin and Cellulose	English adults with excess of weight	40–65	155	[12]
Double-blind, randomized, cross-over intervention study	Chicory-derived inulin (Orafti inulin)	Chicory-derived inulin	Maltodextrin	German healthy men and women having constipation	20–75	54 (33/11)	[13]
Randomized, placebo-controlled, double-blind, cross-over trial	50/50 mixture of oligofructose and inulin; Orafti^®^ Synergy1, Beneo GmbH, Germany)	Inulin-type fructans	Maltodextrin	Norwegian patients with type 2 diabetes	63.1 (mean)	25 (10/15)	[14]
Randomized, double-blind, cross-over design	Chicory inulin (Frutafit supplied by Imperial Suiker-Unie, Sugar Land, TX, USA; produced by Sensus, Roosendaal, The Netherlands)	Low-fiber diet with chicory inulin	Low-fiber control diet	U.S. healthy human subjects	27–49	12 (12/0)	[15]
Four-arm parallel, double-blind, randomized, placebo-controlled trial	Orafti P95, DP 3–9, average DP 4; BENEO-Orafti	OF + maltodextrin/FL	Maltodextrin + FL	U.K. healthy adults	18–50	92 (30/62)	[16]
Multicenter, randomized, placebo-controlled trial performed in obese individuals	Extracted from chicory root, Cosucra, Pecq, Belgium	Inulin and inulin + PA	Maltodextrin and Maltodextrin + PA	Belgian obesity-related metabolic disorder adults	18–65	59	[17]
Randomized, controlled trial	>85% fructo-oligosaccharides; average chain length: seven monomers; Sensus, Roosendaal, The Netherlands	Green tea + Inulin beverage	Placebo beverage	Taiwanese adults with excess of weight	20–50	30	[18]
Randomized, double-blind, parallel, placebo-controlled trial	Synergy 1, namely, inulin/oligofructose 50/50 mix, Orafti, Oreye, Belgium	Inulin + oligofructose 50/50	Maltodextrin	Belgian women with obesity	18–65	30 (0/30)	[19]
Placebo-controlled, double-blind, cross-over trial	Not available	Inulin snack	Control snack	Canadian healthy adults	18–65	48 (22/28)	[20]
Randomized, controlled, cross-over trial	BIOAGAVE agave inulin fiber; Ingredion Incorporated	Inulin-containing chews	control chews	U.S. healthy adults	18–65	29	[21]
Randomized, placebo-controlled trial	Orafti^®^ Synergy 1, Beneo, Tienen, Belgium	Oligofructose-enriched inulin	Maltodextrin	Polish children with celiac disease	4–18	34 (13/21)	[22]
Randomized, placebo-controlled, double-blind, parallel intervention	Fibruline^®^ Instant, Cosucra Group Warcoing, Warcoing, Belgium	Inulin + maltodextrin	Maltodextrin	Danish adults with obesity or excess of weight	18–60	86 (31/54)	[23]
Randomized, single-blind, multicentric, placebo-controlled trial	Extracted from chicory root, Cosucra, Belgium	Inulin	Maltodextrin	Belgian adults with obesity	18–65	110	[24]
Randomized, controlled clinical trial	Not available	Inulin	Maltodextrin	Female patients with type 2 diabetes mellitus	>18	49 (0/49)	[25]
Multicentric, single-blind, placebo-controlled trial	Extracted from chicory root, Cosucra, Belgium	Inulin	Maltodextrin	Belgian patients with obesity	>18	24	[26]
Cross-over randomized, controlled trial	Not available	Inulin	Glucose	Spanish patients with excess of weight	18–65	25 (12/13)	[27]
Randomized, double-blind, placebo-controlled, cross-over study	Orafti^®^ Synergy1–50:50 inulin to fructo-oligosaccharide mix; Beneo GmbH	Inulin-type fructan	Maltodextrin	New Zealand healthy adults	19–65	33 (13/20)	[28]
Double-blind, placebo-controlled study	FrutafitTEX!, Sensus, Roosendaal, The Netherlands	Chicory long-chain inulin	Glucose	Caucasian old patients	55–80	26 (18/8)	[29]
Interventional prospective controlled study	Not available	Low-protein diet and inulin	Low-protein diet	Italian patients with chronic kidney disease	18–80	41 (25/16)	[30]
Double-blind, randomized, placebo-controlled, cross-over study	Bayer BioScience GmbH, Hermannswerder, Potsdam, Germany	VLCI	Maltodextrin	UK healthy adults	20–42	31	[31]
Randomized, double-blind, placebo-controlled, cross-over study	Orafti Synergy, Beneo, Belgium	Inulin	Maltodextrin	U. S. individuals undergoing HD	55 (mean)	12 (6/6)	[32]
Randomized, double-blind, cross-over design	Fibruline Instant; Cosucra Group Warcoing	Inulin	Maltodextrin	Swiss healthy adult women	18–40	32 (0/32)	[33]
Randomized, controlled trial	Chicory root	Inulin	Baseline	U. S. healthy young adults	17–29	174	[34]
Randomized, triple-blind, controlled trial	Chicory root	Inulin	Baseline	Iranian type 2 diabetic women	20–65	49 (0/49)	[35]
Double-blind, placebo-controlled, intervention study	Chicory root	Inulin and oligofructose (50/50)	Maltodextrin	Belgian women with obesity	18–65	30 (0/30)	[36]
Randomized, triple-blind, controlled trial	Quantum High-Tech Biologicals Co. Ltd., Jiangmen, China	Inulin	Baseline	Chinese healthy adults	18–65	57 (22/35)	[37]
Randomized, controlled trial	Not available	Inulin	Baseline	Czechs Patients with type 2 diabetes	18–65	27	[38]
Randomized, controlled trial	Chicory root	Inulin and Arabinoxylan (50/50)	Maltodextrin	U.K. healthy adult men	19–55	20	[39]
Prospective single-arm study	Inulin Biosciences Company, Wuhan, China	Inulin	Baseline	Chinese adult patients with prediabetes	37–69	49 (16/39)	[40]
Three-arm parallel, placebo-controlled, randomized, double-blind study	Jerusalem artichoke	Inulin + fruit juice	Fruit juice	U.K. healthy adults	18–55	66 (33/33)	[41]
Simple randomized intervention study	Frutafit^®^ IQ, Roosendaal, The Netherlands	Inulin	Baseline	Kuwaiti adult women with obesity	18–65	12	[42]
RCT	Not available	Inulin + pomegranate juice	Pomegranate juice	Chinese adults with obesity	18–65	67 (33/34)	[43]
Randomized, controlled trial	Jerusalem artichoke	Inulin	Maltodextrin	Thai children with obesity	7–15	143	[44]

**Table 2:** Effects of inulin on metabolic disturbances and gut microbiota according to its origin, dose, and intervention duration.

Source	Effect on Gut Microbiota	Effect on Metabolic Disturbances and SCFAs	Dose (g/d)	Time (Weeks)	Reference
Chicory	**Increase**: *Bifidobacterium*, *Anaerostipes* **Decrease**: *Bilophila* **No effect**: *Akkermansia*, *Eubacterium*, *Faecalibacterium*, *Lactobacillus*	None	12	4	[13]
Chicory	**Increase**: Total anaerobes, *Lactobacillus* **Decrease**: None **No effect**: *Clostridium*, *Bifidobacterium*, *Enterobacteriaceae*	None	20	6	[15]
Chicory	**Increase**: *Bifidobacterium*, *Anaerostipes*, *B. angulatum* **Decrease**: *Clostridium sensu stricto* **No effect**: *B. adolescentis*, *B. bifidum*	**Increase**: Plasma AST levels **Decrease**: Body weight, BMI, liver stiffness, TC **No effect**: None	16	12	[17]
Chicory	**Increase**: *B. bifidum*, *B. longum B. adolescentis*, *Catenibacterium* **Decrease**: *Desulfovibrio*, *Roseburia*, **No effect**: None	**Increase**: Insulin HOMA-IR, HOMA-ISI **Decrease**: Body weight, BMI **No effect**: HDL-C, LDL-C, HOMA-IR, TGs, TC, SBP, DBP	16	12	[24]
Chicory	**Increase**: *Bifidobacterium*, *Anaerostipes*, *Catenibacterium* **Decrease**: *Actinomyces*, Erysipelotrichaceae, Lachnospiraceae, Enterobacteriaceae **No effect**: None	**Increase**: Linolenic acid **Decrease**: None **No effect**: SCFAs, body weight, BMI, fat mass, waist, SBP, DBP, TC, LDL-C, HDL-C, TG	16	12	[26]
Chicory	**Increase**: *B. angulatum*, *B. ruminantium*, *B. adolescentis* **Decrease**: None **No effect**: None	**Increase**: None **Decrease**: None **No effect**: SCFAs	8	9	[29]
Chicory	**Increase**: None **Decrease**: None **No effect**: *Bifidobacterium*, *Faecalibacterium*	**Increase**: None **Decrease**: None **No effect**: Butyrate, propionate, acetate	10 or 15	13	[32]
Chicory	**Increase**: *Bifidobacterium* **Decrease**: None **No effect**: None	**Increase**: Lactate **Decrease**: Fecal pH **No effect**: Butyrate, propionate, fumarate, acetate, iron absorption	20	4	[33]
Chicory	**Increase**: *B. longum*, *B. adolescentis*, *Anaerostipes hadrus* **Decrease**: None **No effect**: None	**Increase**: Total SCFA **Decrease**: None **No effect**: None	20	4	[34]
Chicory	**Increase**: *A. hadrus*, *B. faecale*, *Bacteroides caccae* **Decrease**: *Ruminococcus faecis*, *Blautia obeum*, *Blautia faecis* **No effect**: None	**Increase**: Insulin sensitivity, IL-10 **Decrease**: Fasting insulin, IL-8 **No effect**: SCFAs, body weight, food intake	20	6	[9]
Jerusalem artichoke	None	**Increase**: FFMI **Decrease**: BMI-z, FMI, LDL-C **No effect**: TC, HDL-C, TGs, FPG, SBP	13	24	[11]
Agave	**Increase**: *B. adolescentis*, *B. breve*, *B. longum*, *B. pseudolongum* **Decrease**: *Desulfovibrio*, *Lachnobacterium* *, *Ruminococcus* * No effect: *B. animalis*, *B. bifidum*, *Akkermansia*, *Faecalibacterium*, *Coprococcus*	**Increase**: None **Decrease**: None **No effect**: Propionate, butyrate, acetate	5 or 7 *	12	[21]
Global artichoke	**Increase**: *Bifidobacterium*, lactobacilli–enterococci **Decrease**: *Bacteroides–Prevotella* **No effect**: *Escherichia coli*, *Eubacterium rectale–Clostridium coccoides group*, *Ruminococcus*	**Increase**: None **Decrease**: None **No effect**: SCFAs	10	6	[31]
Not available	None	**Increase**: HDL-C **Decrease**: Serum insulin, TC, TGs **No effect**:	19	24	[30]
Not available	None	**Increase**: None **Decrease**: FBS, HbA1c, fasting insulin, HOMA-IR, hs-CRP, TNF-α **No effect**: None	10	8	[25]
Not available	**Increase**: *Bifidobacterium*, *Cellulomonas*, *Nesterenkonia* *, *Brevibacterium* * Decrease: *Ruminococcus*, *Dorea* **No effect**: *Lachnospira*, *Oscillospira*	**Increase**: None **Decrease**: None **No effect**: SCFAs	3 or 7 *	12	[20]
Not available	None	**Increase**: None **Decrease**: BMI, body weight, fasting glucose, HbA1c, **No effect**: Fasting insulin, HOMA-IR	10	8	[35]
Not available	**Increase**: *Bifidobacterium*, *Eubacterium*, **Decrease**: *Ruminococcus* **No effect**: None	**Increase**: Butyrate, propionate **Decrease**: None **No effect**: Acetate	10	16	[37]
Not available	**Increase**: *Bifidobacterium*, *Faecalibacterium*, *Akkermansia*, *Anaerostipes* **Decrease**: *Bacteroides* **No effect**: None	**Increase**: Insulin sensitivity, butyrate, propionate **Decrease**: None **No effect**: Acetate	10	12	[38]
Not available	**Increase**: *Bifidobacterium*, *Lactobacillus*, *Anaerostipes* **Decrease**: None **No effect**: None	**Increase**: TGs **Decrease**: HDL-c, LDL-c **No effect**: None	15	24	[40]
Not available	None	**Increase**: None **Decrease**: Body weight, BMI **No effect**: None	21	6	[42]
Jerusalem artichoke	**Increase**: *Bifidobacterium*, *Faecalibacterium*, **Decrease**: None **No effect**: None	**Increase**: None **Decrease**: None **No effect**: Acetate, butyrate, propionate	13	24	[44]

“None” means either that the study showed no outcomes or results were considered irrelevant. * Exclusive results obtained at 7 g/d dose. LDL-C: low-density lipoprotein cholesterol; HDL-C: high-density lipoprotein cholesterol; TC: total cholesterol; TG: triglycerides; FFMI: free fat mass index; FMI: fat mass index; SBP: systolic blood pressure; DBP: diastolic blood pressure; HOMA-IR: Homeostatic Model Assessment of Insulin Resistance; SCFA: short-chain fatty acids; TNF-α: tumor necrosis factor alpha. AST: aspartate aminotransferase; HOMA-ISI: homeostasis model assessment of insulin sensitivity index; BMI-z: body mass index z-score; FPG: fasting plasma glucose; hs-CRP: high-sensitivity c-reactive protein; HbA1c: hemoglobin A1c.

**Table 3:** Effect of inulin combined with other compounds on human metabolism and microbiota according to its origin, dose, and time of intervention. “None” means either that the study showed no outcomes or results were considered irrelevant.

Combination	Effect on Gut Microbiota	Effect on Metabolic Disturbancesand SCFAs	Dose (g/d)	Time (Weeks)	Reference
Inulin–propionate ester	**Increase**: *Bacteroides uniformis*, *Bacteroides xylanisolvens* **Decrease**: *Blautia obeum*, *Eubacterium ruminantium*, *A. hadrus*, *B. faecale*, *Prevotella copri* **No effect**: *Bifidobacterium*	**Increase**: Insulin sensitivity, adipose tissue insulin resistance **Decrease**: Fasting insulin **No effect**: SCFAs, body weight, food intake	20	6	[9]
Inulin–propionate ester	None	**Increase**: PYY and GLP-1 **Decrease**: IHCL, Weight gain, intra-abdominal adipose tissue distribution **No effect**: None	10	24	[12]
Glucose + inulin	None	**Increase**: Acetate, propionate, and butyrate **Decrease**: None **No effect**: GLP-1, PYY, Grhelin	99	3	[27]
Catechins + inulin	None	**Increase**: None **Decrease**: Body weight, fat mass, BMI, blood pressure, glucose, **No effect**: Waist, hip, HDL-C, LDL-C, TGs, TC	534 mg catechins + 11.7 g inulin	3	[18]
Inulin + maltodextrin	**Increase**: *Parabacteroides*, *Bifidobacterium* **Decrease**: *Bilophila*, *Ruminococcus* **No effect**: None	**Increase**: None **Decrease**: Insulin, SBP, DBP, white blood cells **No effect**: BMI, body weight, fat mass, waist, hip, TC, HDL-C, LDL-C, TGs	20	12	[23]
Oligofructose-enriched inulin	**Increase**: *Bifidobacterium* **Decrease**: None **No effect**: None	**Increase**: Acetate, propionate, valerate **Decrease**: None **No effect**: Isovalerate, isobutyrate, butyrate	10	12	[22]
Oligofructose-enriched inulin	**Increase**: *Bifidobacterium*, **Decrease**: *Coprococcus*, *Dorea*, *Ruminococcus* **No effect**: None	**Increase**: None **Decrease**: None **No effect**: SCFAs	16	3	[28]
Oligofructose-enriched inulin	**Increase**: *Bifidobacterium*, *Faecalibacterium prausnitzii* **Decrease**: *Bacteroides intestinalis*, *B vulgatus* **No effect**: None	**Increase**: None **Decrease**: None **No effect**: BMI, body weight, waist/hip ratio, HbA1c, fasting glycemia, insulinemia, TC, HDL-C or LDL-C, and TG	16	12	[36]
Oligofructose-enriched inulin	**Increase**: *B. adolescentis*, *B. longum*, *B. pseudocatenulatum* **Decrease**: None **No effect**: None	**Increase**: None **Decrease**: Acetate, butyrate **No effect**: Isobutyrate and isovalerate	16	12	[19]
Oligofructose-enriched inulin	**Increase**: *B. adolescentis*, *B. longum*, *Bacteriodes vulgatus*, *Faecalibacterium prausnitzii* **Decrease**: *Roseburia* sp., *Eubacterium eligens*, *B. bifidum*, *Anaerostipes butyraticus* **No effect**: *Actinomyces*	**Increase**: None **Decrease**: Body weight **No effect**: BMI, hist, waist, IL-6, HOMA-IR, insulin,	8	16	[10]
Inulin + Arabinoxylan	**Increase**: *Bifidobacterium*, *Propionibacterium* **Decrease**: None **No effect**: None	**Increase**: Acetate **Decrease**: None **No effect**: Butyrate, propionate	8	12	[39]
Inulin + fruit juice	**Increase**: *Bifidobacterium*, *Lactobacillus* **Decrease**: *Eubacterium* **No effect**: None	None	10	3	[41]
Inulin + pomegranate	**Increase**: *Bifidobacterium*, *Akkermansia*, **Decrease**: *Lachnospira*, *Klebsiella* **No effect**: None	**Increase**: None **Decrease**: None **No effect**: Body weight, BMI	10	3	[43]

“None” means either that the study showed no outcomes or results were considered irrelevant. GLP-1: Glucagon-like Peptide-1; PYY: Peptide YY; IHCL: Intrahepatic lipid content.
